# Multicenter outbreak of plasmid-encoded *bla*_OXA-72_ *Acinetobacter baumannii* in southern Poland

**DOI:** 10.1128/aac.00039-26

**Published:** 2026-06-26

**Authors:** T. Kasperski, R. Arazo del Pino, K. Xanthopoulou, T. Burgwinkel, M. Pomorska-Wesołowska, J. Wójkowska-Mach, A. Chmielarczyk, P. G. Higgins

**Affiliations:** 1 Department of Microbiology, Faculty of Medicine, Jagiellonian University Medical College, Krakow, Poland; 2 Institute for Medical Microbiology, Immunology and Hygiene, Faculty of Medicine and University Hospital Cologne, University of Cologne 650804, Cologne, Germany; 3 German Center for Infection Research (DZIF), Partner Site Bonn-Cologne, Cologne, Germany; 4 Department of Microbiology, Analytical and Microbiological Laboratory of Ruda Slaska, KORLAB NZOZ, Ruda Slaska, Poland; Universita degli Studi di Roma La Sapienza, Rome, Italy

**Keywords:** *Acinetobacter baumannii*, carbapenem resistance, *bla*_OXA-72_, *bla*_OXA-23_, *bla*_OXA-1223_, international clone 2 (IC2), GR2 plasmid, whole-genome sequencing (WGS), core genome MLST (cgMLST)

## Abstract

Carbapenem-resistant *Acinetobacter baumannii* (CRAb) causes hard-to-control healthcare outbreaks, but the mobile resistance determinants that sustain regional spread are often underrecognized. We characterized 70 CRAb isolates from 11 hospitals in southern Poland (2019–2022) using whole-genome sequencing and core genome multilocus sequence typing (cgMLST), with reconstruction of carbapenemase context and plasmid structure. All isolates belonged to international clone 2 (IC2; ST2, Pasteur scheme) or to ST425 (*n* = 47), ST195 (*n* = 19), ST208 (*n* = 2), and ST348 (*n* = 1) according to the Oxford scheme. *In silico* KL/OCL typing further differentiated the collection. All isolates were OCL18, whereas the K-locus distribution was dominated by KL125 (*n* = 48) and KL3 (*n* = 19), with two KL2 isolates and one KL9 isolate. *bla*_OXA-72_ was detected in 50/70 isolates (71.4%), indicating an atypically high regional dominance. The OXA-72-encoding gene was carried on the same ~10.9 kb GR2/repAci1 plasmid (p398AB) in all isolates, which was sequence-identical across centers and contained two oppositely oriented copies of *bla*_OXA-72_. Transformation of p398AB into susceptible *A. baumannii* ATCC17978 conferred a carbapenem-resistant phenotype. cgMLST identified a large multicenter cluster of *bla*_OXA-72_-positive isolates spanning multiple cities and hospitals, supporting inter-facility dissemination of a successful IC2 lineage, together with a conserved resistance plasmid. The strict conservation of p398AB during regional spread provides a practical target for rapid molecular screening in outbreak investigations. In contrast, *bla*_OXA-23_ and a novel *bla*_OXA-23_-like variant (*bla*_OXA-1223_) were confined to a minority of isolates. Overall, these data highlight an unusual dominance of plasmid-encoded *bla*_OXA-72_ in a multicenter IC2 outbreak and underscore the need for surveillance integrating clonal relatedness with tracking of transferable resistance plasmids.

## INTRODUCTION

*Acinetobacter baumannii* is a gram-negative, opportunistic pathogen that is often responsible for a variety of healthcare-associated infections. These infections include ventilator-associated pneumonia, bloodstream infections, wound infections, urinary tract infections, and meningitis, particularly affecting critically ill patients in intensive care units (ICUs). The World Health Organization has categorized *A. baumannii* as a priority 1 critical pathogen due to its significant resistance to multiple antibiotics, especially carbapenems (1, 2).

The global spread of carbapenem-resistant *A. baumannii* (CRAb) poses a severe public health threat, leading to high morbidity and mortality rates. In 2019, the U.S. Centers for Disease Control and Prevention classified CRAb as an urgent public health threat, highlighting its role in approximately 8,500 hospital-associated infections and 700 deaths annually in the United States alone (3).

In Europe, the European Antimicrobial Resistance Surveillance Network (EARS-Net) reported an increase in carbapenem-resistant *Acinetobacter* spp. occurrence in 2020–2021 and a slight decrease in 2023. A total incidence of bloodstream infections with carbapenem resistance phenotype of *Acinetobacter* was 2.98 per 100,000 population (4, 5). More than two-thirds (67.2%) of the invasive *Acinetobacter* spp. isolates reported by EU/EEA countries to EARS-Net for 2023 were resistant to at least one of the antimicrobial groups under surveillance (fluoroquinolones, aminoglycosides, and carbapenems). The highest EU/EEA population-weighted mean AMR percentage in 2023 was reported for fluoroquinolones (42.4%), followed by carbapenems (40.1%), and aminoglycosides (36.7%).

The inter-country range in AMR of *A. baumannii* remains one of the widest among all pathogens included in EARS-Net (in 2023, it ranged from 0.0% to 96.6% depending on the reporting country). In Poland, carbapenem resistance reached 82.7% in 2021 (4, 5). *A. baumannii* is naturally resistant to multiple antimicrobials, including penicillins, chloramphenicol, and ertapenem (6). Furthermore, the increase in multidrug-resistant strains has necessitated the increased use of colistin in combination therapy as a last-resort antimicrobial agent. As a result, this selection pressure has facilitated the emergence and global spread of colistin-resistant pathogens (7).

The recent surge in CRAb infections is thought to result from excessive antibiotic use and the consequent antibiotic selection pressure, insufficient infection prevention measures, environmental persistence, and efficient transmission through contact with contaminated hospital surfaces and medical devices (8, 9).

Among the resistance mechanisms of *A. baumannii*, the production of carbapenemases—particularly OXA-type enzymes—plays a pivotal role in conferring carbapenem resistance. The *bla*_OXA-23_ and *bla*_OXA-72_ genes, the latter belonging to the *bla*_OXA-24/40_-like family, are among the most frequently identified in clinical isolates and substantially limit therapeutic options (10–12). The OXA-24/40-like family was first represented by OXA-24, initially described in Spain by Bou et al., whereas OXA-72 was first reported in Asia by Poirel et al. and has subsequently disseminated worldwide (13, 14). In addition to *bla*_OXA-23_ and *bla*_OXA-72_, other OXA-type carbapenemase-encoding genes, such as *bla*_OXA-58_, *bla*_OXA-143_, and *bla*_OXA-235_, have been reported in *A. baumannii* isolates from various geographic regions (15–18). Some variants of the intrinsic *bla*_OXA-51_-like gene can also contribute to carbapenem resistance and are linked to insertion sequences promoting overexpression (19).

Beyond OXA-type enzymes, metallo-β-lactamases, particularly those encoded by *bla*_NDM_, have been increasingly identified among clinical *A. baumannii* isolates. Recent epidemiological studies have demonstrated a significant rise in the global prevalence of *bla*_NDM_-producing strains (20). The emergence and dissemination of *bla*_NDM_ among *A. baumannii* isolates across Europe, including Poland, further limit therapeutic options (20–22).

To combat the spread of CRAb, whole-genome sequencing (WGS) has emerged as a vital tool in understanding the genetic basis of antimicrobial resistance and tracking the transmission dynamics of this pathogen. WGS provides comprehensive insights into the genetic makeup of bacterial isolates, helping to identify resistance genes, plasmid structures, and clonal relationships, which are essential for developing effective infection control strategies (23, 24).

This study focuses on the genomic analysis of a multicenter outbreak of CRAb in southern Poland, to elucidate the genetic diversity and transmission dynamics of CRAb within this multicenter outbreak, highlighting the significance of *bla*_OXA-72_ and other resistance genes in the spread of these CRAb.

## MATERIALS AND METHODS

### Bacterial isolates

A total of 70 CRAb isolates were collected from 11 hospitals in southern Poland between 2019 and 2022 (from 11 cities, mainly Sosnowiec, Czeladź, and Katowice). The isolates were obtained from various clinical samples, including 40 from blood, 26 from urine, and 4 from cerebrospinal fluid (CSF), from patients hospitalized in both ICU and non-ICU settings, including departments of general surgery, orthopedics, neurology, internal medicine, vascular surgery, and nephrology.

### Antimicrobial susceptibility testing

Antibiotic susceptibility testing was performed using the automated MIDITECH Analyser v.12, evaluating a panel of antimicrobial agents including ampicillin/sulbactam, piperacillin/tazobactam, cefoperazone/sulbactam, sulbactam/durlobactam, imipenem, meropenem, ciprofloxacin, amikacin, gentamicin, tobramycin, tigecycline, trimethoprim/sulfamethoxazole, cefiderocol, and colistin. Results were interpreted following EUCAST guidelines (version 15.0, January 2025). Interpretation for cefiderocol was performed according to the Polish document, which is a commentary and guide to the EUCAST recommendations: “Working Group Guidance on Frequently Asked Questions on Applying EUCAST Recommendations” (25). Cefiderocol-resistant strains were defined as <17 mm zones, and susceptible strains were defined as ≥21 mm zones. Tigecycline and sulbactam-durlobactam susceptibility was assessed for all isolates using a gradient diffusion method (Liofilchem Diagnostics, Roseto degli Abruzzi, Italy), and minimum inhibitory concentrations (MICs) were interpreted based on the document mentioned above. A tigecycline MIC > 0.5 was interpreted as resistant. For sulbactam-durlobactam, an MIC ≥ 16 was interpreted as resistant, with MIC ≤ 4 as susceptible. Interpretations for ampicillin/sulbactam and piperacillin/tazobactam were performed according to the Clinical and Laboratory Standards Institute (CLSI) criteria. Due to the absence of EUCAST and CLSI breakpoints, the susceptibility to cefoperazone/sulbactam was evaluated based on the manufacturer’s recommendations and using the ATCC 43498 reference strain as a quality control. Strains resistant to cefoperazone/sulbactam were defined as zone diameter breakpoints ≤15 mm and susceptible zones >21 mm. Strains with intermediate sensitivity were counted as resistant strains.

### Carbapenem resistance genes PCR detection

A multiplex-PCR targeting the most prevalent carbapenemase-encoding genes was used as an initial screen: *bla*_VIM_, *bla*_KPC_, *bla*_NDM_, *bla*_OXA-23_, *bla*_OXA-40_, *bla*_OXA-48_, *bla*_OXA-58_, *bla*_IMP_, *bla*_GES_, *bla*_GIM_, *bla*_IMI_, and IS*Aba1–bla*_OXA-51_ (26).

### WGS

#### Short-read sequencing

Genomic DNA was extracted from the *A. baumannii* isolates using the DNeasy UltraClean Microbial Kit (Qiagen, Hilden, Germany), following the manufacturer’s protocol. Genomic DNA libraries for short-read sequencing were prepared as previously described (27).

WGS was conducted on the Illumina MiSeq platform (Illumina, San Diego, CA, USA). Paired-end sequencing reads with a read length of 2 × 250 bp were generated.

#### Long-read sequencing

High-molecular weight genomic DNA was extracted using the Wizard Genomic DNA Purification Kit (Promega, Madison, WI, USA). The quality and quantity of the DNA were evaluated using a Qubit 4 Fluorometer (Thermo Fisher Scientific, Waltham, MA, USA). Long-read sequencing was performed using the MinION platform (Oxford Nanopore Technologies, Oxford, UK). Libraries were prepared with the Ligation Sequencing gDNA—Native Barcoding Kit 24 V14 (SQK-NBD114.24). The prepared DNA library was loaded onto R10.4.1 flow cells (FLO-MIN114). Basecalling and assembly of the sequencing reads were performed using BugSeq with Flye, an online platform that provides bioinformatic analysis for nanopore sequencing data, allowing for high-accuracy basecalling and *de novo* assembly of genomic sequences (28).

### Plasmid assembly and plasmid transformation

Genomes and plasmids were assembled using a hybrid assembly approach, Unicycler (version 0.4.9), that combined both short reads from Illumina MiSeq and long reads from Oxford Nanopore MinION. Additionally, to confirm whether the *bla*_OXA-72_ gene was plasmid-encoded, plasmid extraction and transformation experiments were performed. Plasmid DNA was isolated from selected *bla*_OXA-72_-positive *A. baumannii* isolates using the QIAprep Spin Miniprep Kit (Qiagen, Hilden, Germany), following the manufacturer’s instructions.

Electrocompetent *A. baumannii* ATCC 17978 cells were prepared and transformed via electroporation as previously described and selected on Luria-Bertani (LB) agar supplemented with 50 µg/mL ticarcillin (29). After transformation, cells were incubated in LB broth for recovery and subsequently plated on LB agar supplemented with 50 µg/mL ticarcillin. Colonies that grew on selective medium were subjected to plasmid DNA extraction, followed by multiplex PCR targeting carbapenemase genes (CARBA PCR) to verify the presence of the *bla*_OXA-72_.

### Plasmid validation by PCR

To confirm whether the *bla*_OXA-72_ gene is plasmid-encoded in isolate 398, PCR assays were performed both on genomic DNA and plasmid DNA from this isolate. Genomic DNA was extracted using the DNeasy UltraClean Microbial Kit (Qiagen). Plasmid DNA was isolated using the QIAprep Spin Miniprep Kit (Qiagen).

Both DNA extracts (genomic and plasmid) were subjected to OXA multiplex PCR (30). The OXA multiplex PCR targets *bla*_OXA-40/72_ and *bla*_OXA-51_-like genes, among others. In addition, a unidirectional PCR using the *bla*_OXA-40/72_ forward primer from the multiplex assay of Cerezales et al. (26) was used to assess the orientation and copy number of *bla*_OXA-72_. When two copies are arranged in opposite orientations on a circular plasmid, the same forward primer can anneal to both copies and generate an amplicon spanning the intervening sequence. Based on the final p398AB sequence, the expected amplicon size was approximately 4.1 kb, consistent with duplication of *bla*_OXA-72_ in opposite orientations on the plasmid.

### Data analysis

The raw sequencing reads underwent quality control, where adapter sequences and low-quality bases were removed. High-quality reads were then assembled *de novo* using the SKESA assembly model (version 2.4.0) integrated within the SeqSphere+ software suite (Ridom GmbH, Münster, Germany). The bioinformatics tools ResFinder (31) and ISfinder (32) were used for the initial screening of acquired resistance genes and insertion sequences, respectively. For the expanded revision analyses, resistance determinants, selected resistance-associated point mutations, and virulence-associated loci were extracted from the SeqSphere+ metadata export based on the NCBI AMRFinderPlus and *A. baumannii* VFDB modules. Oxford and Pasteur MLST were assigned *in silico* using the PubMLST schemes. Because *in silico* Oxford typing of *A. baumannii* can be confounded by the *gdhB* paralogue (*gdhB2*), artifactual *gdhB2-*derived allele calls were excluded before final sequence type assignment, as recommended by Gaiarsa et al. (33). Capsular locus (KL) and outer-core locus (OCL) types were assigned using Kaptive-compatible *A. baumannii* reference databases (34, 35).

### Core genome multilocus sequence typing

The isolates were typed using core genome multilocus sequence typing (cgMLST) as previously described (36). The resulting cgMLST data were used to construct a minimum spanning tree to visualize phylogenetic relatedness. For visualization of closely related groups, isolates differing by ≤10 alleles were provisionally assigned to the same cluster.

### Statistical methods

Descriptive statistics were used to summarize the data set: categorical variables as counts and analyses were performed in IBM SPSS Statistics (IBM Corp., Armonk, NY, USA).

## RESULTS

A total of 70 *A. baumannii* isolates from patients hospitalized in a variety of wards were analyzed in this study (Table 1). Isolates were recovered from 30 female (43%) and 40 male (57%) patients. Ages ranged from 22 to 97 years (mean 66, median 68), with Q1 at 57.25 years, Q3 at 75.75 years, and an IQR of 18.5 years.

**TABLE 1:** Distribution of *A. baumannii* isolates according to sample type and hospital ward^*a*^

The origin of the isolates, total *n* = 70	ICU, *n* = 27	Non-ICU, *n* = 43	
		Surgery, *n* = 15	Non-surgery, *n* = 28
Blood, *n* = 40 (57%)	19 (70%)	8 (53%)	13 (46%)
Urine, *n* = 26 (37%)	6 (22%)	6 (40%)	14 (50%)
CSF, *n* = 4 (6%)	2 (8%)	1 (7%)	1 (4%)

^*a*^ ICU, intensive care unit; non-ICU, non-intensive care unit; CSF, cerebrospinal fluid.

### Antimicrobial susceptibility testing

All *A. baumannii* isolates were resistant to cefoperazone/sulbactam, imipenem, meropenem, ciprofloxacin, and levofloxacin, while no colistin-resistant and sulbactam-durlobactam-resistant isolates were detected. Tigecycline resistance was observed in 99% of isolates. No statistically significant differences in resistance patterns were observed between isolates from blood, urine, and cerebrospinal fluid samples for any of the tested antimicrobials (Table 2).

**TABLE 2:** Distribution of antimicrobial resistance among *A. baumannii* isolates^*a*^

Antibiotic classes	Antimicrobial	Resistant isolates; number (%)			
		Total, *n* = 70	The origin of the isolates		
			Blood, *n* = 40	Urine, *n* = 26	CSF, *n* = 4
Penicillin	Ampicillin/sulbactam Piperacillin/tazobactam	61 (87%) 70 (100%)	33 (83%) 40 (100%)	24 (92%) 26 (100%)	4 (100%) 4 (100%)
Cephalosporins	Cefoperazone/sulbactam Cefiderocol	70 (100%) 4 (5.7%)	40 (100%) 2 (5%)	26 (100%) 2 (7.7%)	4 (100%) 0 (0%)
Carbapenems	Imipenem Meropenem	70 (100%) 70 (100%)	40 (100%) 40 (100%)	26 (100%) 26 (100%)	4 (100%) 4 (100%)
Fluoroquinolones	Ciprofloxacin Levofloxacin	70 (100%) 70 (100%)	40 (100%) 40 (100%)	26 (100%) 26 (100%)	4 (100%) 4 (100%)
Aminoglycosides	Amikacin Gentamicin Tobramycin	65 (93%) 51 (73%) 65 (92%)	38 (95%) 28 (70%) 37 (93%)	23 (88%) 20 (77%) 24 (92%)	4 (100%) 3 (75%) 4 (100%)
Tetracyclines	Tigecycline	69 (99%)	39 (98%)	26 (100%)	4 (100%)
Miscellaneous agents	Colistin Trimethoprim/sulfamethoxazole Sulbactam/durlobactam	0 (0%) 69 (99%) 0 (0%)	0 (0%) 39 (98%) 0 (0%)	0 (0%) 26 (100%) 0 (0%)	0 (0%) 4 (100%) 0 (0%)

^*a*^ CSF, cerebrospinal fluid.

### Carbapenem resistance gene PCR detection

By PCR, *bla*_OXA-40_-like genes were detected in 50/70 isolates (71.4%), whereas *bla*_OXA-23_-like genes were detected in the remaining 20/70 isolates (28.6%).

When analyzed by infection type, *bla*_OXA-40_ genes were most frequently found in blood isolates (31/40), followed by urine (16/26) and CSF (3/4). In contrast, *bla*_OXA-23_-like genes were detected in 10/26 urine isolates, 9/40 blood isolates, and 1/4 CSF isolates. Among blood-derived isolates, the difference between *bla*_OXA-40_-like and *bla*_OXA-23_-like detection was not statistically significant (chi-square = 1.69, df = 1, *P* = 0.193). Detailed isolate-level carbapenemase assignments are provided in Table S1.

### Prevalence and distribution of *bla*_OXA_ genes

Consistent with the multiplex PCR results, *in silico* resistance-gene screening detected the *bla*_OXA-40_-like variant *bla*_OXA-72_ in 50 isolates and *bla*_OXA-23_ in 16 isolates.

Four isolates had a *bla*_OXA-23_-like variant with 99.87% nucleotide identity to *bla*_OXA-23_. Analysis of the amino acid sequence revealed a tyrosine (Y) to phenylalanine (F) substitution at a conserved position 4 within the signal peptide region, defining a novel variant designated *bla*_OXA-1223_, which was deposited in GenBank under accession number NG_242192.1.

### Expanded resistome and selected virulence-associated loci

Expanded genomic analysis showed a conserved multidrug-resistant background across the 70 CRAb isolates (Table S2). All isolates carried the intrinsic *bla*_OXA-66_ gene and the quinolone resistance-associated substitutions GyrA S81L and ParC S84L. The acquired carbapenemase profile comprised *bla*_OXA-72_ (*n* = 50), *bla*_OXA-23_ (*n* = 16), and the novel *bla*_OXA-1223_ variant (*n* = 4). Additional β-lactam resistance determinants included *bla*_ADC-30_ (*n* = 47), *bla*_ADC-73_ (*n* = 19), and *bla*_TEM-1_ (*n* = 41). Aminoglycoside resistance determinants were highly prevalent, including the intrinsic *ant*(3″)-IIa (*n* = 70), *aph*(3″)-Ib (*n* = 69), *aph*(6)-Id (*n* = 69), and the acquired *aac*A16 (*n* = 48), and sulfonamide resistance genes *sul*2 and *sul*1 were detected in 64 and 52 isolates, respectively. To keep the presentation concise and epidemiologically informative, the resistome is summarized here by major antimicrobial classes and key determinants, whereas the isolate-level matrix is provided in Table S2.

Subgroup-specific differences were also apparent (Table S3 and Table 3). The *bla*_OXA-72_-positive isolates were mainly associated with *bla*_ADC-30_, *aac*A16, *sul*1/*sul*2, and frequently *bla*_TEM-1_, whereas *bla*_OXA-23-like_ isolates, including the *bla*_OXA-1223_ subgroup, more often carried *bla*_ADC-73_, *arm*A, *aph*(3′)-VIb, *fts*I A515V, and, in a subset, the *mph*(E)-*msr*(E) macrolide module.

**TABLE 3:** Integrated genomic summary of the major acquired carbapenemase subgroups, including Oxford MLST, KL/OCL assignment, and key accompanying resistance determinants^*a*^

Subgroup	*n* (%)	Oxford ST	KL/OCL	Key co-determinants
*bla*_OXA-72_	50 (71.4)	ST425 dominant; single ST348/ST208; 1 ST425/ST208	KL125 dominant; rare KL2/KL9; all OCL18	*bla*_ADC-30_, *aac*A16, *sul*1*/sul*2, frequent *bla*_TEM-1_
*bla*_OXA-23_	16 (22.9)	ST195 dominant; 1 ST208	KL3 dominant; 1 KL2; all OCL18	*bla*_ADC-73_, *bla*_TEM-1_, a*rm*A, *aph*(3′)*-VIb*, *sul*2, FtsI_A515V; *mph(*E*)-msr(*E*)* subset
*bla*_OXA-1223_	4 (5.7)	ST195	KL3; OCL18	*bla*_ADC-73_, *arm*A, *mph(*E*)-msr(*E*)*, FtsI_A515V, *qac*E*delta*1

^*a*^ KL, capsular locus; OCL, outer-core locus. Detailed isolate-level data are provided in Tables S1–S4.

Virulence-associated loci were largely conserved across the collection (Table S4). Nearly all isolates carried the *csu* pilus cluster, *bfm*RS, *pga*ABCD, *omp*A, *plc/plc*D, and the *bas/bau* iron-acquisition loci, consistent with preserved biofilm-associated and siderophore-related potential in the outbreak population. In contrast, *bap* was present in only 26/70 isolates.

### cgMLST of CRAb isolates

**Fig 1:**
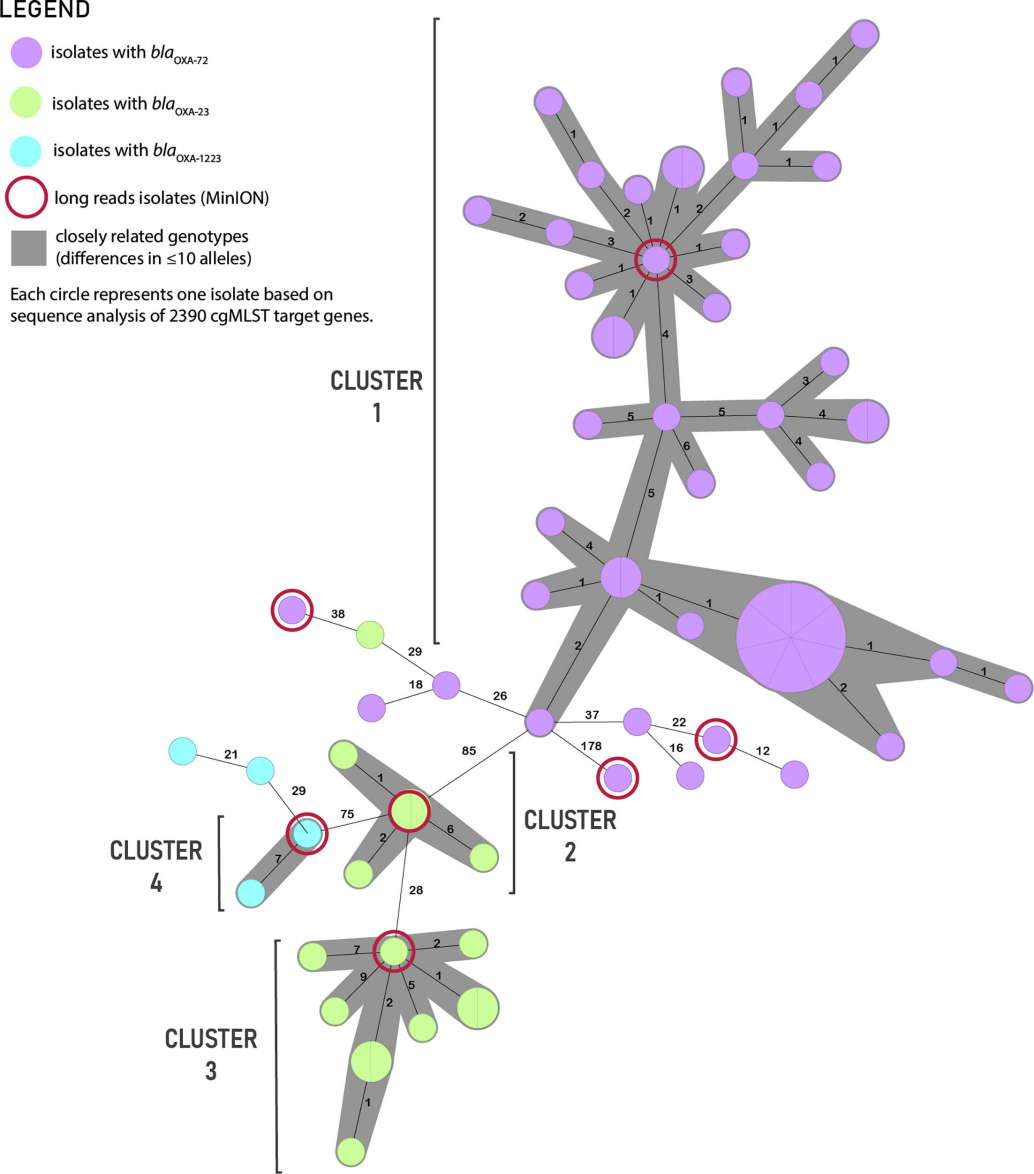
Minimum spanning tree of 70 CRAb isolates generated using Ridom SeqSphere+. Circle size is proportional to the number of isolates represented by each node.

All 70 carbapenem-resistant *A. baumannii* isolates were assigned to international clone 2 (IC2), corresponding to sequence type ST2 according to the Pasteur sequence typing (MLST) scheme. Additional Oxford MLST resolved the collection into ST425 (*n* = 47), ST195 (*n* = 19), ST208 (*n* = 2), and ST348 (*n* = 1), one isolate remained ambiguous between ST425 and ST208 because two *gpi* alleles matched equally well in the draft assembly. cgMLST analysis (Fig. 1 and Table S1) of these isolates revealed 4 phylogenetic clusters and 11 singletons. Cluster 1 was the largest, comprising 42 isolates, spanning five different cities (Czeladź, Katowice, Kłobuck, Sosnowiec, and Tarnowskie Góry). These isolates were predominantly associated with *bla*_OXA-72_ and were mostly derived from blood (*n* = 28, 66.7%), followed by urine (*n* = 11, 26.2%) and CSF (*n* = 3, 7.1%). In terms of hospital departments, isolates from Cluster 1 were most frequently recovered from ICU (*n* = 18, 42.8%), surgical wards (*n* = 10, 23.8%) and internal medicine wards (*n* = 8, 19.0%), with additional isolates from nephrology, rehabilitation, and other non-surgical wards.

The remaining clusters were smaller and more localized. Cluster 2 (*n* = 5) was identified between May 2019 and January 2020 in two cities and was associated exclusively with the *bla*_OXA-23_ gene. Most strains in this cluster came from internal medicine wards (*n* = 4, 80.0%), and urine dominated (*n* = 4, 80.0%). Cluster 3 (*n* = 10) comprised isolates from two cities collected over a longer time span, associated with *bla*_OXA-23_. This group included six isolates from blood (60.0%) and seven from the ICU (70.0%). Cluster 4 (*n* = 2) included strains identified as carrying the novel *bla*_OXA-1223_ variant (*n* = 2, 100%) and originated from distinct departments (laryngology and internal medicine). One isolate was from blood and one from urine.

The singletons (*n* = 11) included eight *bla*_OXA-72_-positive isolates, one *bla*_OXA-23_-positive isolate, and two *bla*_OXA-1223_-positive isolates. These isolates were distributed across eight different cities and various wards, with four associated with blood and seven with urine.

*In silico* KL/OCL typing further differentiated the collection. All isolates were assigned to OCL18, whereas the K-locus distribution was dominated by KL125 (*n* = 48) and KL3 (*n* = 19), with two KL2 and one KL9 isolate. An integrated subgroup-level overview of acquired carbapenemases, Oxford MLST, KL/OCL, and key resistance determinants is provided in Table 3.

A detailed summary of each cluster, including the number of cities, acquired carbapenemase genes, clinical material, and distribution across ward types, is provided in Table 4.

**TABLE 4:** Characteristics of cgMLST-defined clusters among 70 carbapenem-resistant *A. baumannii* isolates, including sample types, carbapenemase genes, cluster, no. of cities, and ward distribution^a^

Cluster no.	Acquired carbapenemase	*N* (%), total *n* = 70	No. of cities	*N* (%), blood	*N* (%), urine	*N* (%), CSF	Ward
1	*bla*_OXA-72_	42 (60%)	5	28 (67%)	11 (26%)	3 (7%)	ICU; 18 (42.8%), surgery; 10 (23.8%), non-surgery; 14 (33.3%)
2	*bla*_OXA-23_	5 (7.1%)	2	1 (20%)	4 (80%)	0 (0%)	Non-surgery; 4 (80%), ICU; 1 (20%)
3	*bla*_OXA-23_	10 (14.3%)	2	6 (60%)	3 (30%)	1 (10%)	ICU; 7 (70%), non-surgery; 2 (20%), surgery; 1 (10%)
4	*bla*_OXA-1223_	2 (2.9%)	2	1 (50%)	1 (50%)	0 (0%)	Non-surgery; 2 (100%)
Singletons	*bla*_OXA-72_ (*n* = 8), *bla*_OXA-23_ (*n* = 1), *bla*_OXA-1223_ (*n* = 2)	11 (15.7%)	8	4 (36%)	7 (64%)	0 (0%)	Non-surgery; 6 (54.5%), surgery; 4 (36.4%), ICU; 1 (9.1%)

^a^ ICU, intensive care unit.

All *bla*_OXA-72_-positive isolates, including those in Cluster 1 and among the singletons, carried the same resistance plasmid. In contrast, the other acquired carbapenemase genes detected in this collection (*bla*_OXA-23_ and *bla*_OXA-1223_) were not plasmid-borne in the sequenced representatives.

To investigate the carbapenemases in more detail, as well as the plasmidome, long-read data were generated and hybrid assemblies were performed with Unicycler, followed by BLAST interrogation in Galaxy. In the *bla*_OXA-1223_-positive isolate, we identified *bla*_OXA-1223_ flanked by two IS*Aba1*-like elements: one located 35 bp upstream of the gene on the positive strand (+) and one approximately 1.6 kb downstream on the opposite strand (−). This arrangement is compatible with a Tn2006-like structure with inverted IS*Aba1* copies, potentially enabling expression of *bla*_OXA-1223_ from the upstream element.

### Plasmid assembly

**Fig 2:**
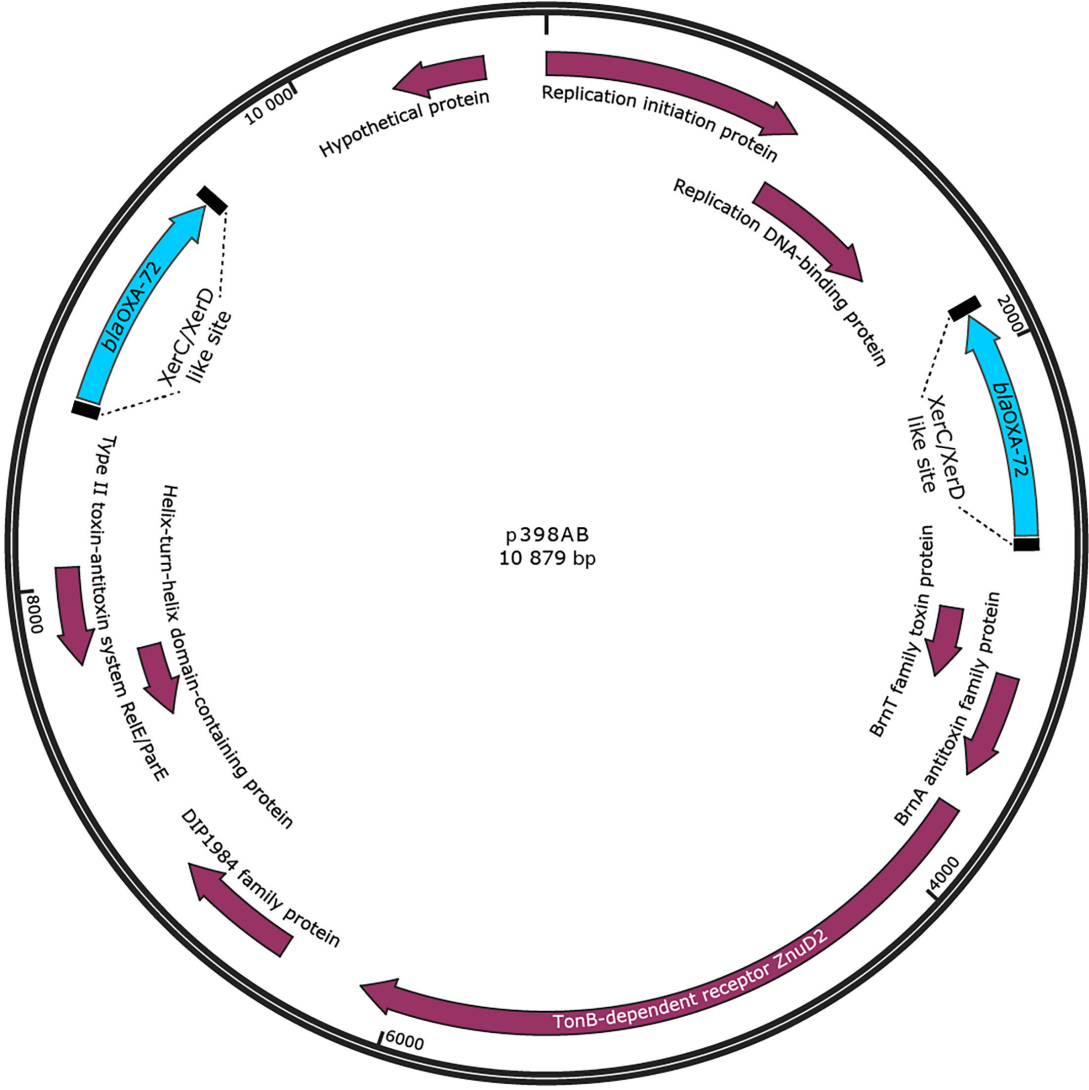
Circular map of the 10,879 bp plasmid p398AB. The figure shows the two oppositely oriented *bla*_OXA-72_ copies, predicted ORFs, the GR2/c-associated replication module, and XerC/XerD-like sites flanking the *bla*_OXA-72_ genes. The opposite orientation of the two *bla*_OXA-72_ copies is consistent with the single-primer PCR assay performed using the *bla*_OXA-40/72_ forward primer from the multiplex of Cerezales et al. (26), which yielded the expected ~4.1 kb amplicon.

Hybrid assembly resulted in the complete reconstruction of a circular plasmid, p398AB, 10,879 bp in length, with a GC content of 34.48%. It was assembled from a Cluster 1 *A. baumannii* isolate carrying *bla*_OXA-72_, and short-read data from all other *bla*_OXA-72_-positive isolates were consistent with the same plasmid sequence, indicating that p398AB is highly conserved and widespread among OXA-72 producers (Fig. 2).

Annotation confirmed the PCR results, with two oppositely oriented *bla*_OXA-72_ copies. Each *bla*_OXA-72_ module was flanked by XerC/XerD-like sites, consistent with the recombination-associated module previously described for pAB120-like OXA-72 plasmids (37, 38). Comparative analysis indicated that p398AB belongs to the same highly conserved family of small 10,879 bp OXA-72 plasmids reported previously in *A. baumannii*.

Six open reading frames ≥ 100 amino acids were predicted, the longest spanning 2,412 bp (804 aa). Plasmid typing identified a replicon belonging to the GR2 group, corresponding to repAci1 in the updated nomenclature (37, 39). This nomenclature is concordant with later rep-based classification frameworks proposed for Acinetobacter plasmids (40, 41).

The same 10,879 bp plasmid structure was found in all other *bla*_OXA-72_-positive isolates (*n* = 50). Hybrid assemblies generated with Unicycler confirmed the presence of an identical plasmid (10,879 bp, GR2 replicon, two *bla*_OXA-72_ copies) across these isolates. Long-read data were generated for four *bla*_OXA-72_-positive isolates (representing both singletons and Cluster 1), one *bla*_OXA-1223_ isolate, and two *bla*_OXA-23_ isolates. In the *bla*_OXA-1223_-positive isolate, long-read-guided analysis identified *bla*_OXA-1223_ flanked by two IS*Aba1*-like elements, one located 35 bp upstream on the positive strand (+) and one ~1.6 kb downstream on the opposite strand (−) (Fig. 2), each approximately 1,180 bp in length and showing >99.7% identity to IS*Aba*1 (AY758396.1), consistent with a Tn2006-like arrangement that may enable expression of *bla*_OXA-1223_ from the upstream copy.

### Plasmid transformation and PCR validation

An OXA multiplex PCR performed on plasmid DNA from *A. baumannii* 398 detected *bla*_OXA-72_, while the intrinsic *bla*_OXA-51_-like gene was absent, confirming the plasmid location of the acquired carbapenemase. Following the transformation of the plasmid into *A. baumannii* ATCC 17978 transformants carried *bla*_OXA-72_ and exhibited a carbapenem-resistant phenotype.

To confirm the copy number and orientation of the gene, a single-primer PCR assay using the OXA-40/72 forward primer from the Cerezales et al. (26) multiplex was performed. This approach generated a 4.1-kb amplicon, consistent with the *in silico* prediction for the circular p398AB sequence and with the presence of two *bla*_OXA-72_ copies in opposite orientations, thereby corroborating the hybrid assembly results.

## DISCUSSION

The present study provides a regionally focused genomic analysis of 70 CRAb isolates recovered from blood, urine, and CSF in southern Poland and confirms that CRAb remains a major challenge in Polish hospitals, particularly in intensive-care settings.

ECDC data from 2023 show a decline in invasive *Acinetobacter* spp. infections to 4.6 per 100,000 population compared with 6.1 per 100,000 in 2021 (4, 5). However, over two-thirds of isolates remained resistant to at least one monitored antimicrobial group (fluoroquinolones, aminoglycosides, or carbapenems). The high resistance levels observed in our study align with trends reported across Europe and in previous analyses of Polish hospitals (4, 5). Serwacki et al. reported that in Poland, the incidence of CRAb infections ranged from 428.6 to 759.5 per 10,000 admissions in ICUs and from 0.3 to 21 per 10,000 in non-ICUs (42).

Nearly 40% of strains in our study originated from ICUs, most recovered from blood. All isolates were phenotypically resistant to carbapenems and fluoroquinolones, and 70% were also aminoglycoside-resistant. Similar findings were reported in our earlier work, with 86% XDR strains and slightly lower levels in 2016–2018 (80% XDR in ICUs) (43). Compared with data from 10–12 years ago, current isolates show higher fluoroquinolone resistance (44, 45). Carbapenem resistance in Poland remains comparable to that observed in southern Europe, particularly Greece and Italy (46, 47). Beyond carbapenems, studies from Greece documented increasing colistin resistance—from 1% in 2012 to 21.1% in 2014 (48), and 27.3% in 2015 (49), while Palmieri et al. reported dissemination of colistin-resistant ST2 clone (50). Although we detected no colistin-resistant strains in 2016–2018, such strains appeared in 2023. Tigecycline resistance in our isolates (99%) aligns with Kasperski et al., who reported 72% resistance in bloodstream isolates (51), whereas Serwacki et al. found higher tigecycline susceptibility among CRAb (77%) (42). In the absence of other therapeutic options, cefiderocol could be a good option; among the strains tested, only 4 isolates showed resistance. Full sensitivity to sulbactam-durlobactam was also demonstrated, but unfortunately, this drug is not registered or available in Poland.

The repertoire of acquired carbapenem-resistance genes in *A. baumannii* is extensive and includes class D β-lactamases (e.g., *bla*_OXA-23-like_, *bla*_OXA-24/40-like_, *bla*_OXA-58-like_, *bla*_OXA-143-like_, and *bla*_OXA-235-like_) and some variants of the intrinsic *bla*_OXA-51-like_, such as *bla*_OXA-82_, as well as class A (*bla*_KPC_) and class B carbapenemases (*bla*_NDM_, *bla*_VIM_, and *bla*_IMP_) (12, 18, 20). Longitudinal data from Poland over 13 years show notable shifts in CRAb genotypes: in 2012, carbapenem resistance was mainly linked to *bla*_OXA-23,_ whereas by 2016, 75% of isolates carried *bla*_OXA-24/40-like_, a trend also observed by Słoczyńska et al. (45, 52, 53). In the present study, PCR screening revealed a predominance of *bla*_OXA-40-like_ (71.4%) and *bla*_OXA-23-like_ (28.6%), with no detection of other carbapenemase genes.

Sequencing confirmed that most recent isolates harbored *bla*_OXA-72_, a member of the *bla*_OXA-24/40-like_ subfamily. Jachowicz-Matczak similarly reported high prevalence of intrinsic *bla*_OXA-66_ (96.8%), acquired *bla*_TEM-1D_ (57.6%), and carbapenemases *bla*_OXA-72_ (71.2%) and *bla*_NDM-1_ (16.0%), supporting the widespread distribution of *bla*_OXA-72_ as well as suggesting IC2 was the prevalent lineage (22). Serwacki et al. found *bla*_OXA-66_ (95%) and acquired *bla*_OXA-40_ (71%) as the most common resistance determinants (42). *Bla*_OXA-72_-positive isolates have also been detected across Europe—including France, Bulgaria, Serbia, and Hungary—within diverse clones (CC78, ST636, ST2, ST492) (54–57).

Interestingly, four isolates in the current study carried a novel *bla*_OXA-23-like_ variant, designated *bla*_OXA-1223_, featuring a Y→F substitution at position 4 within the signal peptide region. Previously (2012), we demonstrated the emergence of *bla*_OXA-82_ from *bla*_OXA-66_ via a Leu167→Val substitution, with overexpression of *bla*_OXA-82_ acting as the sole carbapenem-resistance mechanism in clonally related isolates (58). Although the functional effect of this change was not assessed experimentally, its association with IS*Aba1*, both upstream and downstream mirrors, the Tn2006-like arrangement typically described for *bla*_OXA-23_ and supports the likelihood of promoter-driven expression.

The *bla*_OXA-72_ variant of the *bla*_OXA-40_ family was located on a small GR2/repAci1 plasmid present in two oppositely oriented copies, underscoring the key role of mobile elements in maintaining carbapenem resistance in CRAb. Identical circular plasmids (~10.9 kb, two *bla*_OXA-72_ copies) were detected in Cluster 1 and in *bla*_OXA-72_-positive singletons, indicating regional spread of a highly stable pAB120-like plasmid backbone together with clonal expansion of its host lineages.

These findings point to a large, multi-center CRAb outbreak involving a plasmid harboring two *bla*_OXA-72_ copies. Additionally, two multi-center clusters of *bla*_OXA-23_-positive isolates were identified, highlighting the epidemic potential of CRAb, particularly the IC2 lineage. Together with the strict conservation of p398AB across hospitals and cities, these findings point to a large regional outbreak driven by both clonal dissemination and plasmid stability.

Given the broad hospital distribution of isolates over 2019–2022, repeated transmission from long-term environmental reservoirs or referral-associated inter-facility spread should also be considered.

In a 2025 study, Jachowicz-Matczak et al. described an outbreak associated with an ST600 cluster carrying *bla*_NDM-1_, whereas our data set was dominated by IC2/OXA-72 (22).

Together with reports from Serbia, Hungary, Bulgaria, and other parts of Europe, this pattern suggests that the regional replacement of OXA-23-dominated populations by OXA-24/40-like producers may reflect the successful expansion of particular high-risk lineages and plasmid-gene combinations rather than a single nationwide mechanism.

Our results highlight the importance of comprehensive genomic surveillance and targeted infection control strategies for the detection of multidrug-resistant pathogens. Plasmid-mediated *bla*_OXA-72_ and multicenter spread of CRAb underscore the continuous evolution of antibiotic resistance mechanisms in *A. baumannii* and the urgent need for heightened vigilance and proactive measures in healthcare settings worldwide.

## Data Availability

The raw sequencing reads generated in this project are available at NCBI under BioProject accession number PRJEB104155. The complete hybrid assembly (chromosome and plasmid) is available in GenBank (JBQWDW000000000.1).

## SUPPLEMENTAL MATERIAL

Supplemental tables aac.00039-26-s0001.xlsx Tables S1 to S4.

ASM does not own the copyrights to Supplemental Material that may be linked to, or accessed through, an article. The authors have granted ASM a non-exclusive, world-wide license to publish the Supplemental Material files. Please contact the corresponding author directly for reuse.
